# Supplementary material for: Development of chemokine network inhibitors using combinatorial saturation mutagenesis
Source: Commun Biol. 2025 Apr 3;8:549. doi: 10.1038/s42003-025-07778-6 (PMC11969024; doi:10.1038/s42003-025-07778-6)
Supplement: Supplementary file 4 — Supplementary Data 2 [file 42003_2025_7778_MOESM4_ESM.zip › SupplementaryData3/scripts/xattune_dr.tar!/./script/analysis/JK/JK_E184_CXCL12_ATC_plot.pdf]

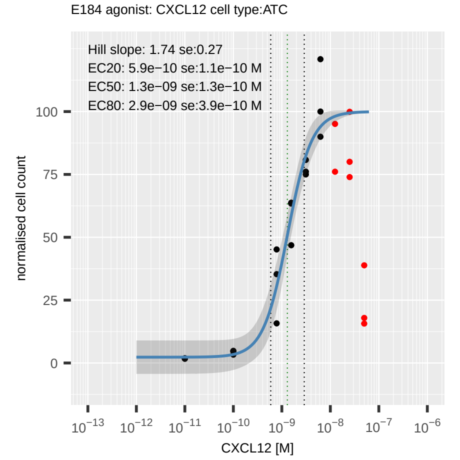

E184 agonist: CXCL12 cell type:ATC
Hill slope: 1.74 se:0.27
EC20: 5.9e−10 se:1.1e−10 M
EC50: 1.3e−09 se:1.3e−10 M
EC80: 2.9e−09 se:3.9e−10 M
normalised cell count
0
25
50
75
100
10^{-13} 10^{-12} 10^{-11} 10^{-10} 10^{-9} 10^{-8} 10^{-7} 10^{-6}
CXCL12 [M]
